# Editorialets

**Published:** 1906-01

**Authors:** 


### Editorialets.

---

**☞TO ADVERTISERS—Lower Half of Second Cover Page Is Available for First Time in Many Years. Price on Request.**

---

Among the doctors who matriculated at the New Orleans Polyclinic last month are: Drs. W. D. Collins, Gustine, Texas; A. S. Pollock, Big Sandy, Texas; W. J. Compton, Crawford, Texas; G. W. Stone, Thornton, Texas; D. D. Swearingen, Groom, Texas; W. E. Davis, Guy's Store, Texas; G. M. Jameson, Buda, Texas, and T. J. Ratliffe, Glory, Texas.

---

Our contract with you is marked T. F. As long as we are advertising in medical journals we would not think of having our advertisement not appear in the famous "Red Back" of Texas. It is, therefore, with pleasure that we ask you to kindly let our contract run on the same as before, *till forbid.*

Wishing you a very happy New Year and trusting that 1906 has in store for you a great abundance of prosperity, we are,

Very truly yours,

Sharpe & Dohme.

---

State Health Officer Tabor and wife have gone to Europe for a two months' trip. Dr. J. H. Florence, the quarantine inspector at Brownsville, is in charge of the office at Austin, and Dr. J. F. Eaves, quarantine inspector at Aransas Pass, takes Dr. Florence's place at Brownsville.

---

Congressman Williams, minority leader, will at once introduce a bill into Congress in accordance with the resolution adopted at Chattanooga by the quarantine convention, turning over international and interstate quarantine to the Federal government.

There is no doubt of the bill passing, as the South has agreed to it all but unanimously, and the North can have no objection; it's not their affair.

---

Dr. Oscar Dowling, editor and publisher of the *Medical Recorder*, Shreveport, La., was elected president of the Tri-State Medical Association (Texas, Arkansas, and Louisiana) at the December meeting at Texarkana.

---

Battle & Co. have just issued the eighth of their series of twelve illustrations of the "Intestinal Parasites," and will send them free to the physicians, on application.

